# Discharge Documentation and Follow-Up of Critically Ill Patients With Acute Kidney Injury Treated With Kidney Replacement Therapy: A Retrospective Cohort Study

**DOI:** 10.3389/fmed.2021.710228

**Published:** 2021-09-14

**Authors:** Xin Yi Choon, Nuttha Lumlertgul, Lynda Cameron, Andrew Jones, Joel Meyer, Andrew Slack, Helen Vollmer, Nicholas A. Barrett, Richard Leach, Marlies Ostermann

**Affiliations:** ^1^Department of Critical Care, King's College London, Guy's and St Thomas' Hospital National Health Service Foundation Trust, London, United Kingdom; ^2^Division of Nephrology, Excellence Centre in Critical Care Nephrology, King Chulalongkorn Memorial Hospital, Bangkok, Thailand; ^3^Critical Care Nephrology Research Unit, Chulalongkorn University, Bangkok, Thailand; ^4^Pharmacy Department, Guy's and St Thomas' National Health Service Foundation Trust, London, United Kingdom

**Keywords:** acute kidney injury, kidney replacement therapies, survival, chronic kidney disease, discharge letter, medication reconciliation, acute dialysis

## Abstract

Leading organisations recommend follow-up of acute kidney injury (AKI) survivors, as these patients are at risk of long-term complications and increased mortality. Information transfer between specialties and from tertiary to primary care is essential to ensure timely and appropriate follow-up. Our aim was to examine the association between completeness of discharge documentation and subsequent follow-up of AKI survivors who received kidney replacement therapy (KRT) in the Intensive Care Unit (ICU). We retrospectively analysed the data of 433 patients who had KRT for AKI during ICU admission in a tertiary care centre in the UK between June 2017 and May 2018 and identified patients who were discharged from hospital alive. Patients with pre-existing end-stage kidney disease and patients who were transferred from hospitals outside the catchment area were excluded. The primary objective was to assess the completeness of discharge documentation from critical care and hospital; secondary objectives were to determine cardiovascular medications reconciliation after AKI, and to investigate kidney care and outcomes at 1 year. The development of AKI and the need for KRT were mentioned in 85 and 82% of critical care discharge letters, respectively. Monitoring of kidney function post-discharge was recommended in 51.6% of critical care and 36.3% of hospital discharge summaries. Among 35 patients who were prescribed renin-angiotensin-aldosterone system inhibitors before hospitalisation, 15 (42.9%) were not re-started before discharge from hospital. At 3 months, creatinine and urine protein were measured in 88.2 and 11.8% of survivors, respectively. The prevalence of chronic kidney disease stage III or worse increased from 27.2% pre-hospitalisation to 54.9% at 1 year (*p* < 0.001). Our data demonstrate that discharge summaries of patients with AKI who received KRT lacked essential information. Furthermore, even in patients with appropriate documentation, renal follow-up was poor suggesting the need for more education and streamlined care pathways.

## Introduction

Acute kidney injury (AKI) is common in the Intensive Care Unit (ICU) affecting more than 50% of critically ill patients (1, 2). Between 10 and 15% of patients with AKI receive kidney replacement therapy (KRT) (3). The development of AKI is associated with serious complications, increased mortality and high health care costs (4–6). Survivors of AKI remain at risk of long-term sequelae, including *de novo* or progressive chronic kidney disease (CKD), end-stage kidney disease (ESKD), cardiovascular events, re-hospitalisation, recurrent AKI, and poorer quality of life, even if renal function initially recovers (7). About 1 in 6 patients with severe AKI become dialysis dependent in the following 2–3 years (8). There is also increasing recognition that an episode of AKI is associated with a deterioration of other chronic illnesses, either directly or as a result of changes to medications (7). Although leading organisations, including the National Institute for Health and Care Excellence (NICE), the Renal Association and the Royal College of General Practitioners (RCGP) recommend nephrology follow-up after an episode of severe AKI, actual follow-up rates range from 8.5 to 41% (3, 9–12). Furthermore, delayed or inadequate follow-up care has been shown to contribute to worse outcomes (12).

A working group of the RCGP United Kingdom (UK) recently published guidance which promotes tailored and timely follow-up care for people who had a hospital admission complicated by AKI. An important component of this recommendation is the safe transition of care between hospital and primary care teams which includes the transfer of relevant information, the importance of accurate discharge documentation, the need for correct coding of the diagnosis of AKI, drug optimisation, and recommendations related to repeat measurement of creatinine and urine protein (13). Repeat measurement of renal function is recommended within 1–12 weeks depending on risk factors and degree of renal recovery after AKI. In addition, it is suggested that high-risk patients are referred for nephrology follow-up, including those with persistent poor kidney recovery and/or glomerular filtration rate (GFR) ≤ 30 ml/min/1.73 m^2^ during follow-up (14). The need for more education, as well as effective communication between healthcare providers and the patient is also highlighted.

Previous reports from centres in France and the United States (US) concluded that hospital discharge summaries were often inadequate to facilitate appropriate follow-up care (15, 16). In the scarcity of data from the UK (17), we aimed to investigate the process and effectiveness of information transfer for critically ill patients with AKI who received KRT and left hospital alive. In particular, we examined the comprehensiveness of the critical care and hospital discharge summaries with regard to AKI diagnosis, the need for KRT, recommendation for follow-up management, and actual kidney and patient outcomes at 1 year. In addition, we explored whether chronic cardioprotective medications were re-started prior to discharge from hospital.

## Materials and Methods

### Setting

Guy's & St Thomas' NHS Foundation Hospital is a tertiary care centre in the UK with a 64-bed, level 2/3 multi-disciplinary adult critical care unit. The critical care unit has a fully computerised electronic patient record system (ICCA, Philips) where all data are recorded at the time of generation. A pertinent summary discharge document is exported in read-only format when the patient is stepped down from critical care to a general or specialist ward. At time of discharge from hospital, the relevant specialty generates the final discharge summary for the primary care team, i.e., general practitioners (GP).

### Study Design and Population

This was a retrospective cohort study. We retrospectively screened the electronic database of all critically ill patients who were admitted to the critical care unit between 1st June 2017 and 31st May 2018. We identified adults (≥16 years old) who had KRT for AKI and left hospital alive. Exclusion criteria were: (a) known ESKD on long-term dialysis or previous renal transplant; and (b) external transfers from ICUs outside the hospital's catchment area. The report was adhered to the STROBE Guideline (18). Patients or the public were not involved in the design, or conduct, or reporting, or dissemination plans of our research.

### Data Collection

Baseline characteristics, comorbidities, chronic medication use, reason for admission, AKI causes, and creatinine values and dialysis status at hospital discharge were collected from the electronic healthcare records. AKI was defined according to the Kidney Diseases: Improving Global Outcomes (KDIGO) Guideline (19). Baseline kidney function was defined as the most recent outpatient non-emergency serum creatinine concentration between 7 and 365 days before admission (19). Estimated glomerular filtration rate (eGFR) was determined using the CKD Epidemiology Collaboration (CKD-EPI) equation (20).

We hand-searched the discharge documents from critical care and the hospital discharge summaries for the documentation of AKI, KRT, and follow-up arrangements, with either these keywords, coding, or a synonymous descriptor. Two investigators (X.Y.C. and N.L.) independently examined all data, and a third investigator (M.O.) adjudicated any differences. One investigator (N.L.) and a team of critical care pharmacists (led by L.C.) independently explored medications prior to ICU admission and at hospital discharge including renin-angiotensin-aldosterone-system inhibitors (RAASi), diabetes drugs, diuretics, and statins. We also identified the frequency and the results of creatinine and urine protein measurements after hospital discharge by checking existing healthcare records, and the outpatient follow-up rates by nephrologists and/or other medical subspecialties within 12 months of hospital discharge. Laboratory results from local hospitals and primary care services were accessed where possible. Finally, we looked at the kidney and patient outcomes, including dependence on chronic dialysis and survival at 1 year. Information about dialysis dependence was obtained from the United Kingdom Renal Registry (UKRR), a mandatory registry which includes all patients with ESKD in the UK.

### Outcomes

The primary outcome was the completeness of the discharge documentation with regard to (1) AKI diagnosis, (2) receipt of KRT, (3) recommendation to monitor renal function, and (4) recommendation to refer for nephrology follow-up. “Completeness” was defined as the inclusion of criteria (1) and (2) and either (3) or (4). Secondary outcomes were proportion of survivors with impaired kidney function (eGFR < 60 ml/min/1.73 m^2^), dialysis dependence at 1 year, survival at 1 year, and rate of follow-up in a specialist nephrology clinic. In addition, we explored whether creatinine and urinary protein results (either by urine dipstick, urine albumin:creatinine ratio, or urine protein:creatinine ratio) were available at 3 months and 1 year after ICU discharge. Chronic kidney disease (CKD) was defined as eGFR persistently < 60 ml/min/1.73 m^2^ at 3 months after hospitalisation (21). Continuation or discontinuation of relevant medications (RAASi, statins, diuretics, diabetes drugs) was explored as an additional outcome.

### Statistical Analysis

Categorical data are presented in numbers and proportions, and continuous data are presented in median (interquartile range, IQR). Comparisons were made using Chi square or Fisher exact test for categorical data and Wilcoxon test for continuous data. Wilcoxon sign rank test was used to assess the difference between eGFR at baseline and 1 year. A *p* < 0.05 was considered statistically significant. Stata 16.1 (StataCorp, Texas, USA) was used for statistical analysis.

## Results

### Patient Characteristics

Between June 2017 and May 2018, 2,380 patients were admitted to the critical care unit of whom 433 (18.2%) critically ill patients received continuous kidney replacement therapy (CKRT) and/or prolonged intermittent kidney replacement therapy (PIKRT) (Supplementary Figure 1). We excluded patients with ESKD (*n* = 96) or a renal transplant (*n* = 22), patients transferred from an ICU in another catchment area (*n* = 72) and patients who died during hospitalisation (*n* = 164). Twelve patients had more than one exclusion criteria.

Ninety-one patients were included in the final analysis of whom 55% were male (Table 1). Their median age was 61 (IQR 47–73) years; 52.8% had pre-existing hypertension, and 25.3% had diabetes mellitus. Twenty-seven (29.7%) patients had prior CKD with a median baseline eGFR of 39 (IQR 27–52) ml/min/1.73 m^2^, of whom 4 (14.8%) were known to nephrology service prior to admission. The main causes of AKI were sepsis (36.3%) and cardiac-related (20.9%).

**Table 1:** Baseline characteristics.

**Variable**	**Value**
Age (years)	61 (IQR 47–73), range 16–90
Men (%)	50 (55)
**Ethnicity (%)**	
- White	68 (74.7)
- Black	14 (15.4)
- Other	9 (9.9)
Diabetes (%)	23 (25.3)
**Comorbidities**	
Chronic kidney disease (%)^*^	
Stage 1 or 2	2 (2.2)
Stage 3	17 (18.7)
Stage 4	6 (6.6)
Stage 5	2 (2.2)
Hypertension (%)	48 (52.8)
Coronary artery disease (%)	15 (16.5)
Active malignancy (%)	14 (15.4)
Atrial fibrillation (%)	13 (14.3)
Congestive heart failure (%)	21 (23.1)
Chronic liver disease (%)	5 (5.5)
Chronic lung disease (%)	9 (9.9)
**Medication use before critical care admission (%)**	
- RAAS inhibitors	35 (38.5)
- Diuretics	25 (27.5)
- Statin	37 (40.7)
- NSAIDs	8 (8.8)
Baseline creatinine (μmol/l)	91 (IQR 73–117), range 46–316
Baseline eGFR (ml/min/1.73 m^2^)	75 (IQR 52–79), range 13–120
**Admission type (%)**	
- Elective surgery	10 (11.0)
- Emergency surgery	13 (14.3)
- Medical	68 (74.7)
**Aetiology of AKI (%)**	
- Sepsis	33 (36.3)
- Post-operative setting	17 (18.7)
- Cardiac related cause	19 (20.9)
- Liver related cause	1 (1.1)
- Hypoperfusion	7 (7.7)
- Obstructive uropathy	3 (3.3)
- Primary renal disease	11 (12.1)

*eGFR, estimated glomerular filtration rate; AKI, acute kidney injury; NSAIDs, non-steroidal anti-inflammatory drugs; RAAS, renin-angiotensin-aldosterone-system; IQR, interquartile range*.

* ***CKD staging:** Stage 1, signs of kidney disease and eGFR ≥ 90 ml/min/1.73 m^2^, Stage 2, eGFR 60–89 ml/min/1.73 m^2^, Stage 3, eGFR 30–59 ml/min/1.73 m^2^, Stage 4, eGFR 15–29 ml/min/1.73 m^2^, Stage 5, eGFR < 15 ml/min/1.73 m^2^ or dialysis*.

### Discharge Documentation

The critical care discharge summary included documentation about AKI in 85.7% of all patients; in 82.4% of cases, it was reported that the patient had received KRT; in 51.6% of summaries, further renal function monitoring was recommended, and 47.3% of summaries included a recommendation for nephrology follow-up (Table 2; Supplementary Figure 2). Overall, only 50 AKI survivors (54.9%) had complete critical care discharge documents.

**Table 2:** Discharge documentation.

	**Record of AKI**	**Record of KRT**	**Recommendation to monitor renal function**	**Recommendation to refer for nephrology follow-up**	**“Complete” discharge summary^a^**
Critical care discharge summary	78 (85.7%)	75 (82.4%)	47 (51.6%)	43 (47.3%)	50 (54.9%)
Hospital discharge summary	65 (71.4%)	56 (61.5%)	33 (36.3%)	19 (20.9%)	29 (31.8%)

*AKI, acute kidney injury; KRT, kidney replacement therapy*.

a *The completeness of the discharge documentation including (1) AKI diagnosis AND (2) Receipt of KRT AND either (3) Recommendation to monitor renal function or (4) Recommendation to refer for nephrology follow-up*.

Hospital discharge summaries included less AKI-related information compared with critical care summaries: AKI was mentioned in only 71.4% documents, “need for KRT” in 61.5%, recommendation for kidney function monitoring in 36.3%, and a recommendation for nephrologist follow-up in only 20.9% cases (Table 2). Only 29 (31.9%) hospital discharge documents included complete information. Patients whose critical care discharge summaries fulfilled the “completeness” criteria for documenting an AKI episode were more likely to also fulfil the “completeness” criteria in the hospital discharge summaries when compared to those critical care discharge summaries that failed to meet the “completeness” criteria.

Patients with poor kidney function or ongoing need for dialysis at time of hospital discharge were more likely to have complete documentation and nephrology follow-up. There was no statistically significant difference in assessment of serum creatinine and proteinuria during the follow-up period between patients with complete and incomplete discharge documents: serum creatinine was measured in 96.6 vs. 87.1% of patients (*p* = 0.26), and proteinuria was determined in 27.6 vs. 19.4% of patients (*p* = 0.38), respectively (Table 3). There was no difference in mortality at 1 year between patients with and without complete discharge documentation (Table 3). Multivariate logistic regression analysis showed that complete discharge documentation was associated with nephrology follow-up when adjusted for baseline CKD status, creatinine at discharge and completeness of critical care discharge documentation [odds ratio (OR) 7.14 (95% confidence interval 1.36–37.40), *p* = 0.02] (Supplementary Table 1).

**Table 3:** Characteristics and outcomes by completeness of discharge documentation.

**Discharge summary**	**Critical Care discharge summary**			**Hospital discharge summary**		
	**Incomplete** **(*n* = 41)**	**Complete** **(*n* = 50)**	***P*-value**	**Incomplete** **(*n* = 62)**	**Complete** **(*n* = 29)**	***P*-value**
**Baseline**						
Age (years)	61 (48, 71)	61 (47, 75)	0.48	63 (47, 71)	57 (48, 75)	0.60
Male gender (%)	23 (46)	27 (54)	0.84	33 (66)	17 (34)	0.63
Diabetes (%)	8 (34.8)	15 (65.2)	0.25	16 (69.6)	7 (30.4)	0.86
Chronic kidney disease (%)	9 (33.3)	18 (66.7)	0.14	15 (55.6)	12 (44.4)	0.09
Hypertension (%)	19 (39.6)	29 (60.4)	0.27	31 (64.6)	17 (35.4)	0.44
Coronary artery disease (%)	5 (33.3)	10 (66.7)	0.40	7 (46.7)	8 (53.3)	0.051
Active malignancy (%)	7 (50)	7 (50)	0.69	11 (78.6)	3 (21.4)	0.54
Atrial fibrillation (%)	6 (46.2)	7 (53.9)	0.93	7 (53.9)	6 (46.2)	0.23
Congestive heart failure (%)	9 (42.9)	12 (57.1)	0.82	14 (66.7)	7 (33.3)	0.89
Chronic liver disease (%)	3 (60)	2 (40)	0.65	4 (80)	1 (20)	1.00
Chronic lung disease (%)	5 (55.6)	4 (44.4)	0.73	7 (77.8)	2 (22.2)	0.71
Baseline creatinine (μmol/l)	89 (72, 105)	91 (74, 132)	0.16	90 (72, 108)	98 (74, 123)	0.32
Baseline eGFR (ml/min/1.73 m^2^)	75 (62, 85)	75 (46, 75)	0.09	75 (62, 79)	66 (49, 75)	0.23
**Admission diagnosis (%)**						
- Surgical	15 (65.2)	8 (34.8)	0.03	16 (69.6)	7 (30.4)	0.86
- Medical	26 (38.2)	42 (61.8)		46 (67.7)	22 (32.4)	
Creatinine at discharge^*^ (μmol/l)	82 (60, 125)	155 (95, 302)	<0.001	86 (64, 155)	205 (118, 328)	0.0003
eGFR at discharge^*^	78 (43, 118)	36 (17, 67)	<0.001	67 (33, 107)	24 (13, 46)	0.0002
Dialysis dependence at discharge (%)	0	7 (14)	0.02	1 (1.6)	6 (20.7)	0.004
**Outcomes**						
Nephrology follow-up (%)	1 (2.4)	21 (42.0)	<0.001	5 (8.1)	17 (58.6)	<0.001
Creatinine measurement (%)	36 (87.8)	46 (92)	0.73	54 (87.1)	28 (96.6)	0.26
Proteinuria measurement (%)	9 (21.95)	11 (22)	1.00	12 (19.4)	8 (27.6)	0.38
Mortality (%)	7 (17.1)	8 (16)	0.89	11 (17.7)	4 (13.8)	0.64

*eGFR, estimated glomerular filtration rate*.

* *In non-dialysis dependent patients*.

### Medication Reconciliation

The proportions of patients who received RAASi, anti-diabetic drugs, diuretics, and statins prior to admission and at hospital discharge are shown in Supplementary Table 2. In patients taking RAASi (*n* = 35) and/or statin (*n* = 37) before admission, the medications at hospital discharge included RAASi in only 20 (57.1%) and statins in 14 (37.8%) of cases. In addition, 12.5 and 1.9% of patients were newly started on RAASi and statin medications during their hospital stay.

### Post-discharge Follow-Up

Only 22 (24%) patients were reviewed in a nephrology clinic within 12 months of discharge. Those who received nephrology follow-up were characterised by worse renal function or being dialysis dependent (*n* = 7) at time of discharge from hospital. They were also more likely to have proteinuria measurement within the following 12 months (40.9 vs. 15.9%, *p* = 0.01) (Table 4). Seventy-six were followed-up by other medical specialties. Overall, in dialysis-independent survivors, creatinine results were available in 88.2 and 71.8% at 3 and 12 months, respectively. Correlation between eGFR at hospital discharge and eGFR at 3 and 12 months was poor [*r*^2^ = 0.62 (*p* < 0.001) and 0.55 (*p* < 0.001), respectively]. Quantitative assessment of proteinuria was performed in only 11.8% of patients at 3 months and in 21.1% at 12 months (Table 5).

**Table 4:** Characteristics and outcomes by nephrology vs. no-nephrology follow-up in following 12 months after hospital discharge.

	**Not seen by nephrologist** **(*n* = 69)**	**Seen by nephrologist** **(*n* = 22)**	***P*-value**
Baseline CKD (%)	17 (24.6)	10 (45.5)	0.06
Known to nephrologists prior to admission (%)	2 (13.3)	2 (20.0)	1.00
Creatinine at hospital discharge (μmol/L)	95 (68–146)	334 (232–458)	<0.001
eGFR at hospital discharge (ml/min/1.73 m^2^)	63 (36–98)	17 (12.5–26)	<0.001
KRT at hospital discharge (%)	0	7 (31.8)	<0.001
**Outcomes**			
Creatinine measurement (%)	60 (87)	22 (100)	0.11
Proteinuria measurement (%)	11 (15.9)	9 (40.9)	0.01

*CKD, chronic kidney disease; eGFR, estimated glomerular filtration rate; KRT, kidney replacement therapy*.

**Table 5:** Outcomes.

	**Discharge from hospital**	**3 months**	***P*-value^#^**	**1 year**	***P*-value^##^**
Death (*n*)	–	1	–	15	–
Survivors (*n*)	91	90	–	76	–
Need for dialysis (*n*)	7	5	–	5	–
**Cr measurement performed^a^** **(%)**	84/84 (100)	75/85 (88.2)	–	51/71 (71.8)	–
Cr value (μmol/l), median (IQR)	117 (72–206)	118 (78–162)	0.11	100 (77–141)	0.04
eGFR (ml/min/1.73 m^2^), median (IQR)	53.5 (24.5–86.5)	55 (36–72)	0.53	55 (38–74)	0.15
eGFR < 60 ml/min/1.73 m^2^ (%)	46/84 (54.8)	39/75 (52)	<0.001	28^b^ / 51 (54.9)	<0.001
**Proteinuria measurement^a^** **(%)**	No data	10/85 (11.8)	No data	15/71 (21.1)	No data

*Cr, creatinine; eGFR, estimated glomerular filtration rate; IQR, interquartile range*.

a *In non-dialysis dependent survivors*.

b *Of 28 patients, 12 (42.9%) had pre-existing CKD, and 16 (57.1%) developed new CKD*.

# *3 months vs. at discharge*.

## *1 year vs. at discharge*.

### One-Year Outcomes

The overall 1-year post-ICU discharge survival rate was 82%. Of those who underwent repeat assessment of renal function during the 12-month period after discharge from hospital, a large proportion (~50%) had significant ongoing renal problems that warranted specialist review/input. Of the 7 patients who were still dialysis dependent at hospital discharge, 3 recovered sufficient kidney function within 12 months to be independent of dialysis, 2 died, and 2 developed ESKD and remained chronically dialysis dependent. Three additional patients became newly dependent on dialysis within 12 months of hospital discharge (Supplementary Figure 1).

In the remaining cohort of patients who were not dialysis-dependent at 1 year and had creatinine results available (*n* = 71), 54.9% had an eGFR < 60 ml/min/1.73 m^2^ consistent with CKD stage 3 or worse compared with 27.2% prior to hospitalisation (*p* < 0.001). In 57.2% of patients with CKD stage 3 or worse at 1 year, there was no evidence of pre-existing CKD. Median eGFR declined significantly from 75 (IQR 52–79) at baseline to 55 (IQR 38–74) ml/min/1.73 m^2^ at 1 year (*p* = 0.0003) (Table 5).

## Discussion

Our study has revealed significant gaps in AKI aftercare in a UK tertiary care setting. In patients who had KRT for AKI and survived, important information relating to AKI, KRT receipt, and follow-up recommendations after hospitalisation was missing in 45% of critical care discharge summaries and almost 79% of hospital discharge letters. Cardioprotective medications which were discontinued during stay in hospital were not re-started before hospital discharge in 40–60% of patients. Despite classifying as high-risk patients, proteinuria was infrequently monitored following discharge from hospital, and only 24% were reviewed by a nephrologist. These findings call for more education and training but also indicate an urgent need for improvement in the systematic information transfer between critical care, hospital teams and primary care providers.

The importance of the above is underscored by the growing evidence that the burden of severe AKI extends beyond the duration of hospitalisation (4). Whilst the majority of our cohort of AKI survivors were liberated from KRT before discharge from hospital, the 1-year mortality was 18% (22). Importantly, in those who survived, in the first year alone, 31% developed *de-novo* CKD and 8% progressed to dialysis dependent ESKD. This further emphasises the urgent need to optimise the aftercare of this high-risk group (23–25) albeit there being ongoing debate and controversy what constitutes optimal aftercare (26–29). Similar to reports in the literature, we found that the proportion of patients who had a nephrology review and/or assessment of creatinine and quantitative proteinuria was low even though 30% of the patients had pre-existing CKD (3, 9, 30). Although multiple factors may play a role, our data suggest that opportunities for improved care are missed, including early recognition of CKD, appropriate initiation of nephroprotective interventions, and timely nephrology referral, all of which are known to prevent CKD progression and reduce the risk of emergency initiation of dialysis and premature death (7, 31).

An accurate written discharge hand-over is a cornerstone of information transfer between healthcare providers and is essential to facilitate continuity of care (32). Although the discharge document is a formal factual report, it is most often produced by a junior member of the medical team who may have had variable input into the patient's care. The reasons for incomplete discharge documentation are likely to be multifactorial, including lack of awareness of the long-term complications of AKI but also a false sense of security in cases where serum creatinine is relatively low (e.g., in patients with muscle wasting) and eGFR is overestimated as also shown by the poor correlation between GFR at discharge and subsequent measurement in our study (3, 33). Of equal importance to the clinical information that a discharge document holds is its use for coding and reimbursement purposes. Failure to record an episode of AKI treated with KRT can have serious implications and affect patients' future or long-term management (34).

Our data suggest that if the discharge document includes a recommendation to arrange a referral to nephrology services, there is a greater likelihood this is acted upon by primary care and a greater chance of subsequent monitoring of kidney function and proteinuria measurement. Unfortunately, the low referral and follow-up rates in our study are similar to other reports in the literature. A previous study reported that whilst 91% of nephrologists agreed that patients who received dialysis for AKI should be followed up (35), nephrology follow-up rates ranged from 6 to 19% in AKI survivors to only 21–50% in those who had acute dialysis within 1 year after hospitalisation (9, 12, 35–39). Nephrology follow-up was found to be associated with reduced mortality (12, 40). The reasons for low referral rates in our study are unclear and need to be further explored.

Medication reconciliation after AKI can be challenging (41). Several observational studies have suggested that RAASi administration after renal recovery may halt CKD progression and reduce long-term mortality, but more confirmatory studies are needed (42–46). The role of statins after AKI remains unclear, too (47). Statin use was associated with reduced 2-year mortality but had no effects on cardiovascular outcomes or CKD progression (48). Our data show that 40–60% of patients who were taking RAASi's and statins pre-hospitalisation were not re-started on these medications before discharge from hospital. A proportion of these discontinuations may have been for valid clinical reasons relating to the evolution of the patient's condition, but it is possible that some were oversights where the medicine was stopped but inadvertently not re-started, and future work should explore this in more depth. As many of these medicines have pleiotropic effects, the impact on control of other conditions including hypertension, diabetes, and cardiovascular disease is unknown.

Our data highlight the gaps in practise and probable lack of awareness about the long-term prognosis of AKI survivors. Given the large number of staff, often junior, and in rotating roles, individual education and training is unlikely to be the best effector of change. Moreover, it is likely that institutions need to develop standardised approaches to recognition, documentation and subsequent management of this patient group, with in-built quality metrics to ensure ongoing improvement in routine patient care, similar to other areas in clinical medicine (17, 49). Incorporation of a structured framework into the discharge document has been recommended (11, 17). In addition to detailing past events, it should include a recommendation for nephrotoxin avoidance, weight and blood pressure monitoring advice, cardiovascular medication management, and sick day rules (28). An automatic AKI follow-up clinic was found to increase the proportion of patients seen by nephrologists and triggered interventions (37). In addition, post ICU recovery clinic may have a role in supporting the GP in the first 3 months after hospital discharge with focused practise on AKI recovery. For example, at our hospital, the post-ICU clinic letter routinely includes information about AKI diagnosis and staging, number of days treated with KRT, and recommendations for general renal management. Once established, iterative quality improvement methodologies should then be employed to confirm impact on patient-centred outcomes allowing robust upscaling and dissemination. Until more evidence is available from ongoing studies (50), the current recommendations by the RCGP should apply to the follow-up management of AKI survivors.

We acknowledge several limitations to our study. First, this was a retrospective observational study with a small sample size and 1-year follow up period. We were unable to show any associations between completeness of discharge documentation and development of relevant patient-centred outcomes, including progression of CKD, risk of mortality, quality of life, need for re-hospitalisation and cardiovascular events. Second, our data represents a single-centre population in the context of the UK healthcare setting and the findings may not be generalisable. However, these results should trigger local audits and reviews in other centres given the consistency of our findings with the existing literature. Third, we focussed on patients with AKI who received KRT since the risk of serious kidney and non-kidney outcomes is highest in this cohort. We are unable to comment on patients with less severe AKI. Fourth, we report a high prevalence of CKD at 1 year but acknowledge that creatinine and urine protein results were not available for all patients. Fifth, it was not possible to explore the exact reasons for incomplete or absent AKI documentation in the discharge summary. Sixth, we compared medications pre- and post-hospitalisation but did not investigate whether the dosages were appropriate when the medication was re-started. Finally, future projects are needed to investigate whether completeness of discharge summaries correlates with patient-centred short- and long-term outcomes. Further studies should also identify which patient subgroups are at highest risk of accelerated deterioration in renal function, thus allowing healthcare providers to prioritise them and to avoid unnecessary interventions in low-risk patients.

This is the first published report describing gaps in the aftercare of ICU patients with severe AKI in a UK hospital, whilst also confirming existing concerns that patients who had KRT for AKI and survived are at high risk of long-term complications. Our study identifies that there is substantial scope for better aftercare, particularly in relation to the information transfer from critical care to non-critical care services and primary care providers, and in monitoring of renal function. We believe that this is likely best achieved through a standardised, iterative, multi-disciplinary quality-improvement approach to advance the care delivered to this important patient group. This will need to be investigated in a future project.

## Data Availability Statement

The original contributions presented in the study are included in the article/Supplementary Material, further inquiries can be directed to the corresponding author/s.

## Ethics Statement

Ethical approval was not provided for this study on human participants because the project had institutional approval (GSTT/10207). The need for Ethics review and individual informed consent was waived as this was a retrospective analysis of data collected prospectively for routine clinical care, and there was no breach of privacy or anonymity (UK National Research Ethics Service). Written informed consent for participation was not required for this study in accordance with the national legislation and the institutional requirements.

## Author Contributions

XYC contributed with data collection and writing the first draught of the manuscript. NL contributed to data collection, statistical analysis, and revision of the paper. LC oversaw medication conciliation and revision of the paper. AJ, AS, HV, NB, and RL interpreted the data and revised the paper. MO originated, supervised the project, and revised the paper. All authors approved the final draught.

## Conflict of Interest

The authors declare that the research was conducted in the absence of any commercial or financial relationships that could be construed as a potential conflict of interest.

## Publisher's Note

All claims expressed in this article are solely those of the authors and do not necessarily represent those of their affiliated organizations, or those of the publisher, the editors and the reviewers. Any product that may be evaluated in this article, or claim that may be made by its manufacturer, is not guaranteed or endorsed by the publisher.
