# Supplementary material for: Discharge Documentation and Follow-Up of Critically Ill Patients With Acute Kidney Injury Treated With Kidney Replacement Therapy: A Retrospective Cohort Study
Source: Front Med (Lausanne). 2021 Sep 14;8:710228. doi: 10.3389/fmed.2021.710228 (PMC8476795; doi:10.3389/fmed.2021.710228)
Supplement: Supplementary file 1 [file Data_Sheet_1.docx]

**Supplementary data**

**Supplementary Figure 1 Flow chart**


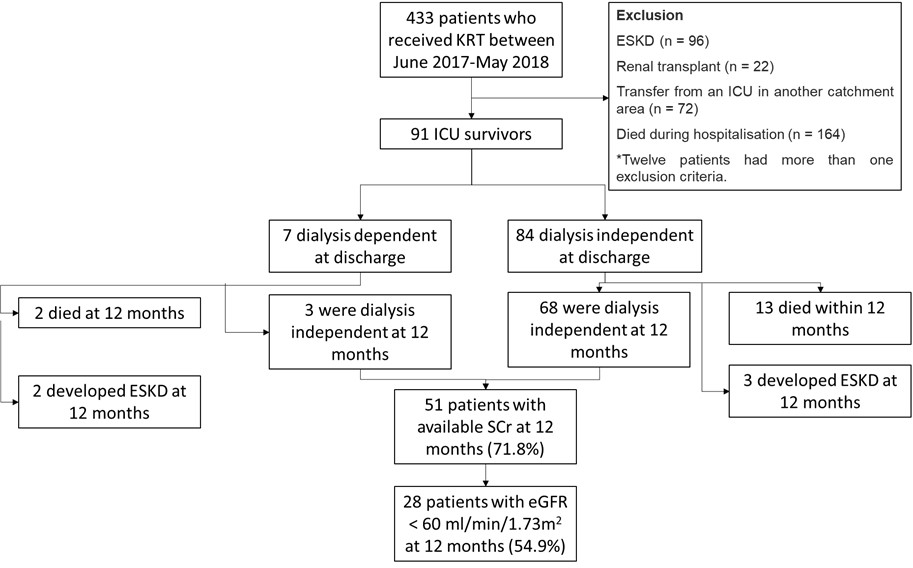


**Abbreviation:** ESKD, end stage kidney disease; eGFR, estimated glomerular filtration rate; ICU, intensive care unit; KRT, kidney replacement therapy; SCr, serum creatinine

**Supplementary Figure 2 Proportions of AKI and KRT records, recommendation for renal function follow-up, and recommendation for nephrology follow-up included in discharge documents**

**
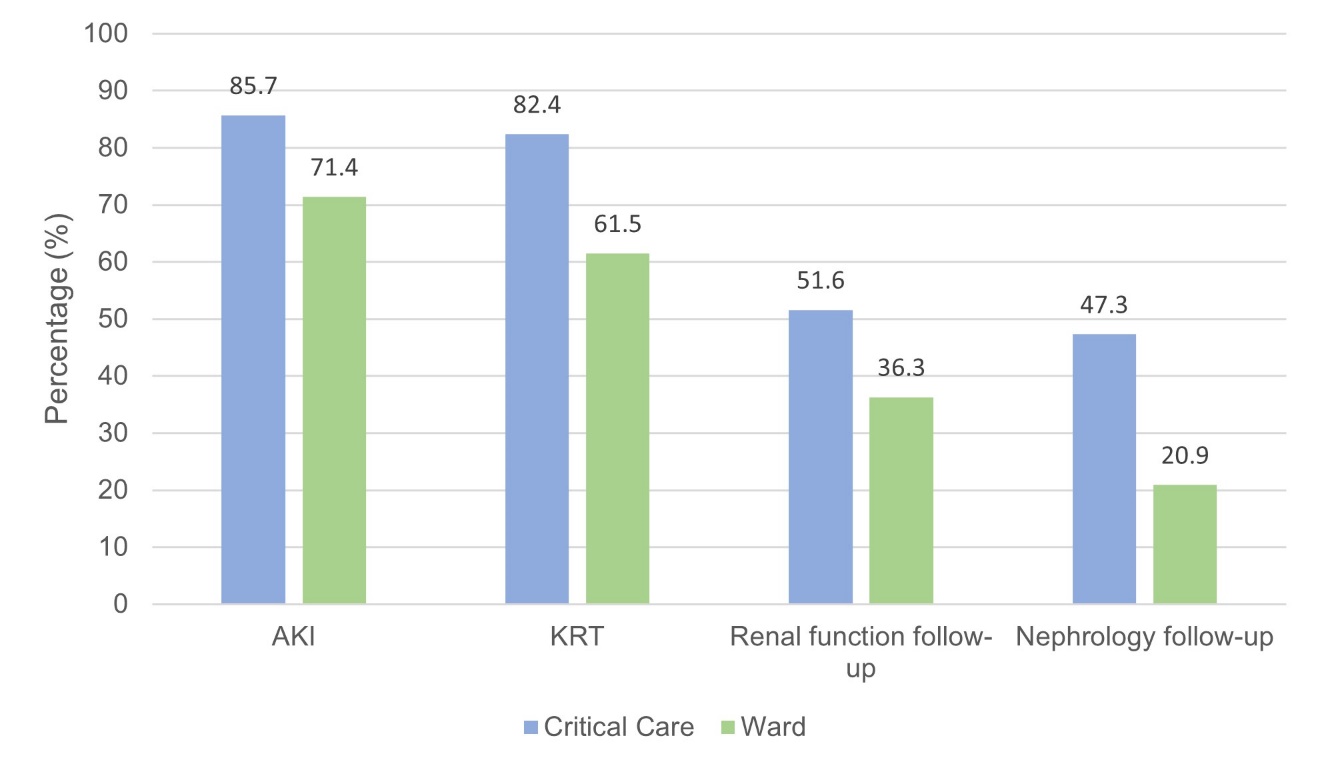
**

**Abbreviations:** AKI, acute kidney injury; KRT, kidney replacement therapy

**blue bars:** critical care discharge letter

**green bars:** hospital discharge summary

**Supplementary Table 1 Multivariate logistic regression for associated factors with nephrology follow-up after hospital discharge**

| **Variables** | **OR** | **95% CI** | **P value** |
| --- | --- | --- | --- |
| Complete hospital discharge documentation | 7.14 | 1.36-37.40 | 0.02 |
| Complete critical care discharge documentation | 3.51 | 0.31-39.69 | 0.31 |
| Baseline CKD status | 1.07 | 0.23-5.06 | 0.93 |
| Serum creatinine at discharge | 1.01 | 1.00-1.02 | 0.001 |

**Abbreviations:** OR, odds ratio; CI, confidence interval; CKD, chronic kidney disease

**Supplementary Table 2 Proportions of RAASi, diabetes drugs, diuretics, and statin which patients received prior to critical care admission and before hospital discharge**

| **Types of medications** | **Received medications before hospitalisation** | | | |
| --- | --- | --- | --- | --- |
|  | **No** | | **Yes** | |
| **Started/continued before discharge** | **No** | **Yes** | **No** | **Yes** |
| RAASi (n=91) | 59 | 7 | 15 | 20 |
| Anti-diabetic drugs (n=91) | 71 | 0 | 7 | 13 |
| Diuretics (n=91) | 61 | 5 | 8 | 17 |
| Statin (n=91) | 53 | 1 | 23 | 14 |

**Abbreviations:** RAASi, renin-angiotensin-aldosterone-system inhibitors
